# Serological Surveillance of Betacoronaviruses in Bat Guano Collectors: Pre-COVID-19 Pandemic and Post-SARS-CoV-2 Emergence

**DOI:** 10.3390/v17060837

**Published:** 2025-06-10

**Authors:** Sasiprapa Ninwattana, Spencer L. Sterling, Khwankamon Rattanatumhi, Nattakarn Thippamom, Piyapha Hirunpatrawong, Pakamas Sangsub, Thaniwan Cheun-Arom, Dominic Esposito, Chee Wah Tan, Wee Chee Yap, Feng Zhu, Lin-Fa Wang, Eric D. Laing, Supaporn Wacharapluesadee, Opass Putcharoen

**Affiliations:** 1 Thai Red Cross Emerging Infectious Diseases Clinical Center, King Chulalongkorn Memorial Hospital, Bangkok 10330, Thailand; 2 Faculty of Medicine, Chulalongkorn University, Bangkok 10330, Thailand; 3 Henry M. Jackson Foundation for the Advancement of Military Medicine, Bethesda, MD 20817, USA; 4 Department of Microbiology and Immunology, Uniformed Services University, Bethesda, MD 20814, USA; 5 Department of Biology, Faculty of Science, Ramkhamhaeng University, Bangkok 10240, Thailand; 6 Protein Expression Laboratory, Frederick National Laboratory for Cancer Research, NIH, Frederick, MD 21701, USA; 7 Infectious Diseases Translational Research Programme, Department of Microbiology and Immunology, Yong Loo Lin School of Medicine, National University of Singapore, Singapore 117545, Singapore; 8 Programme in Emerging Infectious Diseases, Duke-NUS Medical School, Singapore 169857, Singapore; 9 Division of Infectious Diseases, Department of Medicine, Faculty of Medicine, Chulalongkorn University, Bangkok 10330, Thailand

**Keywords:** COVID-19, SARS-CoV-2, betacoronaviruses, zoonotic spillovers, surrogate neutralizing antibodies, binding antibodies

## Abstract

Community-based serosurveillance for emerging zoonotic viruses can provide a powerful and cost-effective measurement of cryptic spillovers. Betacoronaviruses, including SARS-CoV, SARS-CoV-2 and MERS-CoV, are known to infect bats and can cause severe respiratory illness in humans, yet remain under-surveyed in high-risk populations. This study aimed to determine the seroprevalence of betacoronaviruses in an occupational cohort in contact with bats before and after the emergence of SARS-CoV-2. Serum samples from pre- and post-COVID-19 pandemic were screened using antigen-based multiplex microsphere immunoassays (MMIAs) and a multiplex surrogate virus neutralization test (sVNT). Pre-pandemic samples showed no SARS-CoV-2 antibodies, while post-pandemic samples from vaccinated participants displayed binding and neutralizing antibodies against SARS-CoV-2 and a related bat CoV. Furthermore, one participant (1/237, 0.43%) had persistent antibodies against MERS-CoV in 2017, 2018 and 2021 but was seronegative in 2023, despite reporting no history of traveling abroad or severe pneumonia. The observed sustained antibody levels indicate a possible exposure to MERS-CoV or a MERS-CoV-like virus, although the etiology and clinical relevance of this finding remains unclear. Ongoing surveillance in high-risk populations remains crucial for understanding virus epidemiology and mitigating zoonotic transmission risk.

## 1. Introduction

Coronaviruses are enveloped, positive-sense single-stranded RNA viruses belonging to the subfamily *Orthocoronavirinae* in the family *Coronaviridae*. This subfamily *Orthocoronavirinae* is divided into four genera: *Alphacoronavirus*, *Betacoronavirus*, *Gammacoronavirus* and *Deltacoronavirus*. The genus *Alphacoronavirus* consists of two common human coronaviruses (HCoVs), HCoV-229E and HCoV-NL63, which typically cause mild respiratory illnesses. The other two common HCoVs, HCoV-OC43 and HCoV-HKU1, belong to the genus *Betacoronavirus*. This genus also consists of three emerging human coronaviruses: severe acute respiratory syndrome coronavirus 1 (SARS-CoV-1), Middle East respiratory syndrome coronavirus (MERS-CoV) and SARS-CoV-2. SARS-CoV-2 was first detected in December 2019 in Wuhan, China before quickly spreading globally and officially being declared a pandemic in March 2020, causing significant socioeconomic impacts and claiming more than 6.9 million lives before May 2023, when the COVID-19 pandemic was no longer considered a public health emergency of international concern [1].

Bats, specifically insectivorous horseshoe bats (genus: *Rhinolophus*), have been implicated as the probable hosts of multiple betacoronaviruses, including MERS-CoV, SARS-CoV and SARS-CoV-2. SARS-CoV-related coronaviruses (SCr-CoVs), including WIV1, WIV16 and RsSHC014, were detected in Chinese horseshoe bats (*R. sinicus*) [2,3]. Furthermore, previous studies have shown that horseshoe bats are the likely reservoir of SARS-CoV-2, based on phylogenetic analysis [4,5]. SARS-CoV-2-related coronaviruses (SC2r-CoVs) were detected in Asian *Rhinolophus* bats, including RaTG13 (*R. affinis*, China), BANAL-52 (*R. malayanus*, Laos), BANAL-103 (*R. pusillus*, Laos) and RshSTT200 (*R. shameli*, Cambodia) [6,7,8]. In Thailand, a SC2r-CoV virus known as RacCS203 was detected in the *R. acuminatus* bat species in an artificial cave (asbestos cement pipe) in Chachoengsao province [9]. Bat caves in Thailand can be used for many purposes, such as tourism, religious practices, and sometimes bat guano harvesting, where insectivorous bats live inside the caves.

Bat guano is rich in carbon, nitrogen, sulfur and phosphorus content and is widely used as a fertilizer to enhance agricultural productivity. It is harvested weekly from a bat cave in Ratchaburi province, which houses more than three million insectivorous bats. Group C betacoronaviruses, including bat CoV HKU4, bat CoV HKU5 and MERS-like CoV, with 76%, 80% and 77% *RdRp* sequence identity, respectively, were previously detected in dry bat guano collected between 2006 and 2007 from this bat cave [10]. These findings highlight the perceived risk of zoonotic spillover at this human–wildlife interface of viruses, including coronaviruses, which are of significant concern to public health. Bat guano collectors, who are routinely exposed to bat excreta or bat carcasses, are considered at higher risk for zoonotic spillover due to their frequent contact with known viral reservoirs.

In this study, we conducted serological surveillance in a high-risk community to identify evidence of zoonotic spillover of betacoronaviruses. This study employed two serological techniques, an antigen-based multiplex microsphere immunoassay (MMIA) and a multiplex surrogate virus neutralization test (multiplex sVNT), to assess binding and surrogate neutralizing antibodies against both human and bat coronaviruses. Our findings provide evidence for the need for continuous surveillance in high-risk communities.

## 2. Materials and Methods

### 2.1. Ethical Approval, Participant Recruitment and Informed Consent

This research adhered to the principles of the Declaration of Helsinki and was approved by the Institute Review Board of the Faculty of Medicine, Chulalongkorn University, Thailand (No. 380/59, No. 211/64 and No. 0158/66), and all experiments were performed in accordance with relevant guidelines and regulations.

An introductory visit with community leaders and local authorities at the site was conducted to generate interest and obtain permission for study activities before the research commenced. With the obtained approval, community members were informed of study participation opportunities through established communication channels. Interested individuals who met the enrollment criteria were invited, along with parents or legal guardians if aged 12–17 years old, to engage in further discussions with research staff. Adults who provided consent, or children who provided assent with their parent’s or legal’s consent, were enrolled in the study. Participation in the study was completely voluntary, and all participants were informed that they could withdraw from the study at any time without consequences [11].

### 2.2. Human Biological Samples and Data Collection

This study enrolled two participant cohorts. The pre-pandemic cohort included individuals whose samples were collected twice, in 2017 and 2018. The post-COVID-19 emergence cohort comprised participants whose samples were collected twice, in 2021 and 2023. The study was conducted in Ratchaburi province at 4 different time points: in April–May 2017 and June 2018 under the United States Agency for International Development (USAID) Emerging Pandemic Threats PREDICT project, November 2021 and January 2023 under the Centers for Research in Emerging Infectious Diseases Network (CREID) EID-SEARCH project.

With obtained consent, a total of 356 human blood samples (a maximum of 5 mL) and oral, nasal or nasopharyngeal swabs were collected from individuals, either guano collectors working in or community members living in close proximity (within 10 km) to the bat cave in Ratchaburi province. The whole blood samples were then centrifuged, and the serum samples were collected and stored at −80 °C until analysis. The swabs were collected in lysis buffer (BioMerieux, Marcy l’Etoile, France) containing guanidine thiocyanate and stored at −80 °C until analysis.

Two sets of questionnaires were used in this study based on two separate projects. The first set, designed and administered prior to the COVID-19 pandemic, did not include questions related to COVID-19 infection and vaccination status. Standardized questionnaires were administered by study staff to every participant to gain insight information on demographics, livelihood, animal contact history and unusual illness symptoms. All biological samples and questionnaire data were labeled with a unique alphanumeric identification code assigned to each enrolled participant during the consent process. No personal identifying information was recorded on the sample vials or the questionnaires.

### 2.3. Serological Assays

**Figure 1:**
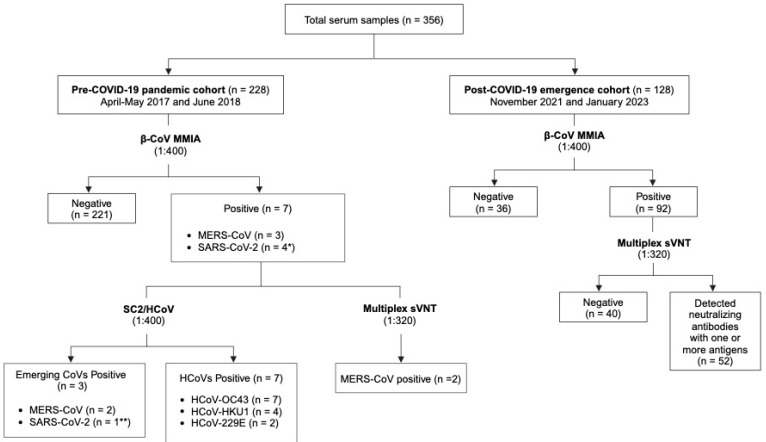
Serological screening of serum samples using MMIA and multiplex sVNT. The pre-pandemic samples positive by the β-CoV MMIA were further tested using the SC2/HCoV MMIA and the multiplex sVNT. Post-COVID-19 emergence samples positive by the β-CoV MMIA were further tested using the multiplex sVNT to determine percent signal inhibition. * Two samples were positive with more than one antigen, with the MFI against SARS-CoV-2 being highest. ** One sample was weakly positive for SARS-CoV-2 (4952 MFI, see Table S1 by the SC2/HCoV MMIA. Created in BioRender (https://BioRender.com).

Three serological assay panels were used in our study (Figure 1). The multiplex microsphere immunoassay (MMIA), designed to capture antibodies targeting the spike glycoproteins of coronaviruses, consisted of two panels: the bat-associated betacoronavirus binding panel (β-CoV MMIA), used as a first-tier screening test, with follow-up screening from preliminary positives on a human coronavirus binding panel (SC2/HCoV MMIA). The multiplex surrogate neutralization test (multiplex sVNT) was also used to detect surrogate neutralizing antibodies from any samples positive by the β-CoV MMIA. Furthermore, the multiplex sVNT was used to screen for surrogate neutralizing antibodies in samples from the post-COVID-19 emergence cohort that were positive by the β-CoV MMIA.

#### 2.3.1. The β-CoV MMIA

All serum samples from pre-pandemic and post-COVID-19 emergence cohorts were screened with the β-CoV MMIA, consisting of commercially produced recombinant spike glycoprotein from three emerging human coronaviruses (SARS-CoV-1, SARS-CoV-2 (WT), MERS-CoV) (Curia, Albany, NY, USA) and five bat coronaviruses (bat CoV RaTG13, bat CoV ZXC21, bat CoV Rs4874, bat CoV HKU9 and bat CoV PDF-2180) with an additional mock antigen, consisting of filtered soluble proteins from HEK cells transfected with the empty pcDNA3.1 vector, which were included in the panel to control for non-specific binding. The design, expression, coupling of spike and mock proteins to Bio-Plex Pro magnetic microspheres (Luminex Corp., Austin, TX, USA), and binding assay have been previously described [12,13,14]. Briefly, serum samples were heat-inactivated at 60 °C for 30 min and diluted in 1X phosphate buffer saline (PBS) to one dilution at 1:400. The diluted sera were added to a 96-well plate containing antigen-coupled beads and incubated with agitation at room temperature for 45 min. After three rounds of washing with washing buffer (1XPBS, 0.05% Tween20, 0.02% Sodium Azide), 100 µL of anti-human IgG diluted 1:5000 in 1XPBS 0.05% Tween20 (PBST) was added to the plates and incubated at room temperature for 45 min with agitation. Next, after three washes, Streptavidin-r-phycoerythrin (Bio-Rad, Hercules, CA, USA) diluted at 1:1000 in PBST was added to each well and incubated at room temperature for 45 min with agitation, and plates were washed three times. Finally, 100 µL PBST was added to the plates and agitated for 10 min before the plates were read on the Bio-Plex 200 HTF multiplexing system (Bio-Rad). Binding antibody levels are reported as median fluorescence intensity (MFI). In the absence of experimentally infected or confirmed naturally acquired infection sera, samples were considered positive with a median fluorescence intensity (MFI) above 4774, as reported elsewhere [14]. Serum samples that showed seropositive for the β-CoV MMIA were further tested using multiplex sVNT. Seropositive pre-pandemic serum samples were additionally tested with the SC2/HCoV MMIA to assess cross-reactivity between HCoVs.

#### 2.3.2. The SC2/HCoV MMIA

This SC2/HCoV MMIA contains seven spike antigens, including the three emerging human coronaviruses (SARS-CoV-1, SARS-CoV-2 (WT), MERS-CoV) and four common HCoVs (HCoV-OC43, HCoV-NL63, HCoV-229E and HCoV-HKU1) [12]. The assay aimed to assess hCoV-associated cross-reactivity between these viruses in pre-pandemic serum samples. This assay used the testing strategy described above, except with the inclusion of a second serum dilution at a concentration of 1:8000.

#### 2.3.3. Multiplex Surrogate Virus Neutralization (sVNT)

A multiplex surrogate virus neutralization (sVNT) assay was performed to further investigate neutralizing antibodies of serum samples previously positive by the binding MMIA. The sVNT assay targeting the receptor-binding domain (RBD) of SARS-CoV-2 (ancestral strain), SARS-CoV-1, bat CoV RaTG13, bat CoV WIV-1, bat CoV Rs2018B, bat CoV RsSHC014 or the spike proteins of MERS-CoV [published elsewhere]. The design of the RBD proteins and antigen coupling processes have been described previously [15,16,17]. The multiplex sVNT protocol was followed as described in a previous publication [15,18]. Briefly, serum samples were heat-inactivated at 60 °C for 30 min. A master mix of RBD-coat beads was incubated with serum samples with a dilution of 1:320 at 37 °C for 60 min with agitation, followed by 50 µL of phycoerythrin-conjugated human angiotensin-converting enzyme 2 (ACE2) and human dipeptidyl peptidase-4 (DPP-4) receptors (2 mg/mL; Genscript), and incubated at 37 °C for 30 min with agitation. Subsequently, plates were washed twice with 1% bovine serum albumin in 1XPBS (assay buffer) and resuspended with assay buffer before obtaining the final readings using Bio-Plex 200 HTF multiplexing system (Bio-Rad). Neutralizing antibody levels were calculated and reported as percent signal inhibition.

$$\%\text{Signal Inhibition}=\left(1-\frac{\text{MFI value of sample}}{\text{MFI value of blank}}\right)\times 100\%$$

The cut-off values have been optimized and updated by the Duke-NUS’s laboratory for the targeted antigens: 44.54% (SARS-CoV-2), 53.75% (bat CoV RaTG13), 46.25% (bat CoV WIV-1), 41.69% (bat CoV Rs2018B), 52.27% (bat CoV RsSHC014) and 34.93% (MERS-CoV).

### 2.4. Conventional Family-Wide PCR Assays to Detect Betacoronavirus RNA

Pre-pandemic oral, nasal and nasopharyngeal swabs from the USAID PREDICT project were previously tested and published by Yadana et al. [11]. For the post-COVID-19 emergence samples collected from participants enrolled in 2021 and 2023, total nucleic acid was extracted from 400 µL of nasopharyngeal swabs in lysis buffer using a MagDEA Dx SV kit (Precision System Science, Chiba, Japan) with the automated magLead 12gC machine (Precision System Science), and 50 µL of total nucleic acid (total NA) was eluted. All collected swab samples were screened for betacoronaviruses using the pan-CoV PCR protocol developed by Quan et al. [19]. Briefly, cDNA synthesis was performed using 8 µL of the total NA with the SuperScript III First-Strand Synthesis System (Thermofisher Scientific, Waltham, MA, USA) according to manufactural’s protocol, followed by the nested PCR targeting RNA-dependent polymerase (RdRp) region. The first-step PCR reaction mixture contained 2 µL of cDNA, 2.5 µL of 10× PCR buffer, 0.75 µL of 50 mM magnesium choline, 0.5 µL of 10 mM dNTP mix, 1 µL of each primer (20 µM), 0.1 µL of Platinum™ Taq DNA Polymerase and nuclease-free water, with a final volume of 25 µL for each reaction. The second-step PCR was performed using nested primers as follows: 1 µL of PCR product, 2.5 µL of 10× PCR buffer, 0.75 µL of 50 mM magnesium choline, 0.5 µL of 10 mM dNTP mix, 1 µL of each primer (20 µM), 0.1 µL of Platinum™ Taq DNA Polymerase and nuclease-free water, with a final volume of 25 µL for each reaction. The thermal cycling profile was applied as described by Quan et al. [19]. Positive and negative controls were incorporated into every assay run to uphold the validity of the tests. Subsequently, the PCR products were visualized using a 2% agarose gel. Further confirmation was achieved through Sanger sequencing of the positive nested amplicons, sending the purified PCR product to the 1st BASE Company (Selangor DE, Malaysia).

### 2.5. Statistical Analysis

Data analysis was performed using R version 4.2.2. The Unpaired Mann–Whitney U test was used to compare the median MFI values of each targeted antigen in the β-CoV MMIA between samples collected in 2021 and 2023. A *p*-value < 0.01 was considered statistically significant.

## 3. Results

### 3.1. Study Population and Samples

All participants consented to complete the main questionnaire and provide biological samples. A total of 356 serum samples and 356 swabs (oral, nasal or nasopharyngeal) were collected from 237 unique healthy participants who were enrolled in the study from April 2017 to January 2023 from a single community site in Ratchaburi province. Among the participants, 78 were enrolled for the study more than once (2–4 times) (Table 1). The samples were collected before the pre-pandemic (*n* = 228) and during the COVID-19 pandemic (*n* = 128). Most of the participants were enrolled only once (159, 67%), 87 were female (55%), and 72 were male (45%).

Enrollment in 2017 was balanced between females (50.44%) and males (49.56%), whereas the majority of participants enrolled in 2018 were female, and in 2021 and 2023, the majority of participants were male (Table 2). Most participants were middle-aged and older adults. More than 80% of participants enrolled in 2018 (93.04%), 2021 (87.27%) and 2023 (83.56%) reported living in the community for more than 10 years. There were 28 and 38 participants reported having bat guano collection as their livelihood (not necessarily their primary livelihood) in 2021 and 2023, respectively. Among the bat guano collectors, more than 90% reported collecting bat guano once a week, and approximately 50–60% had been working at the guano collection site for more than five years. The majority of the bat guano collectors were wearing personal protective equipment (PPE), including masks (71.43% and 52.63%), face covers or parts of t-shirts stretched to the face (75.00% and 78.95%), long-sleeved shirts or overshirts (53.75% and 65.79%) and long pants (53.57% and 65.79%) in 2021 and 2023, respectively (Table S2). All bat guano collectors enrolled in 2021 reported utilizing at least one type of PPE, whereas, in 2023, one participant did not wear any PPE.

### 3.2. Pre-Pandemic Cohort

**Figure 2:**
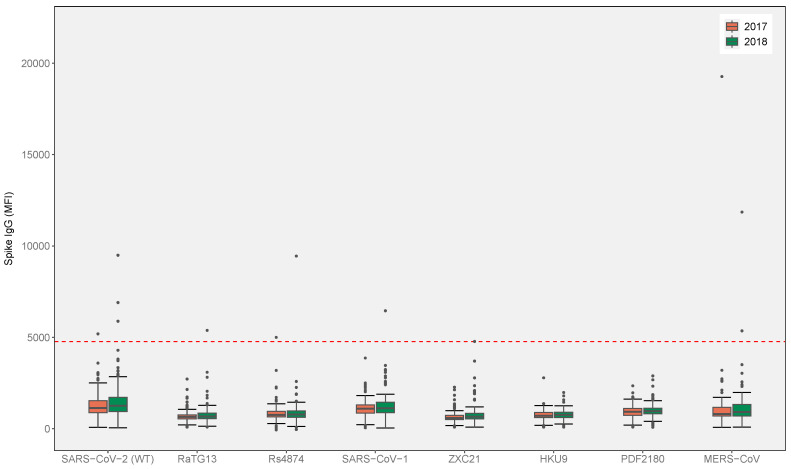
The β-CoV MMIA serological screening of pre-pandemic cohort (*n* = 228). Serum samples collected in 2017 (*n* = 113) and 2018 (*n* = 115) were tested at a 1:400 dilution for IgG reactivity to spike proteins of betacoronavirus to simultaneously detect emerging human coronaviruses and bat coronaviruses. IgG MFI values above the threshold cutoff (4774 MFI, indicated by the red dashed line) were considered seropositive.

A total of 228 pre-pandemic samples were collected two times—in April–May 2017 (*n* = 113) and June 2018 (*n* = 115)—from the same collection site. We detected coronavirus seroreactivity in seven (7/228, 3.07%) samples, including two samples (2/228, 0.88%) that were only reactive to SARS-CoV-2, and two samples (2/228, 0.88%) that were positive for multiple coronaviruses, including SARS-CoV-2 and Rs4874 (1/228, 0.44%), and SARS-CoV-2, RaTG13, SARS-CoV-1 and Rs4874 (1/228, 0.44%) (Figure 2). Additionally, we detected three (3/228, 1.32%) samples with reactive antibodies against MERS-CoV (Figure 2). These three serum samples were collected from two participants who worked as a forest ranger (*n* = 2, collected in 2017 and 2018 with strong MFI; 19,269 and 11,855, respectively; Table S3) and a bat guano collector (*n* = 1, collected in 2018, 5352 MFI).

**Figure 3:**
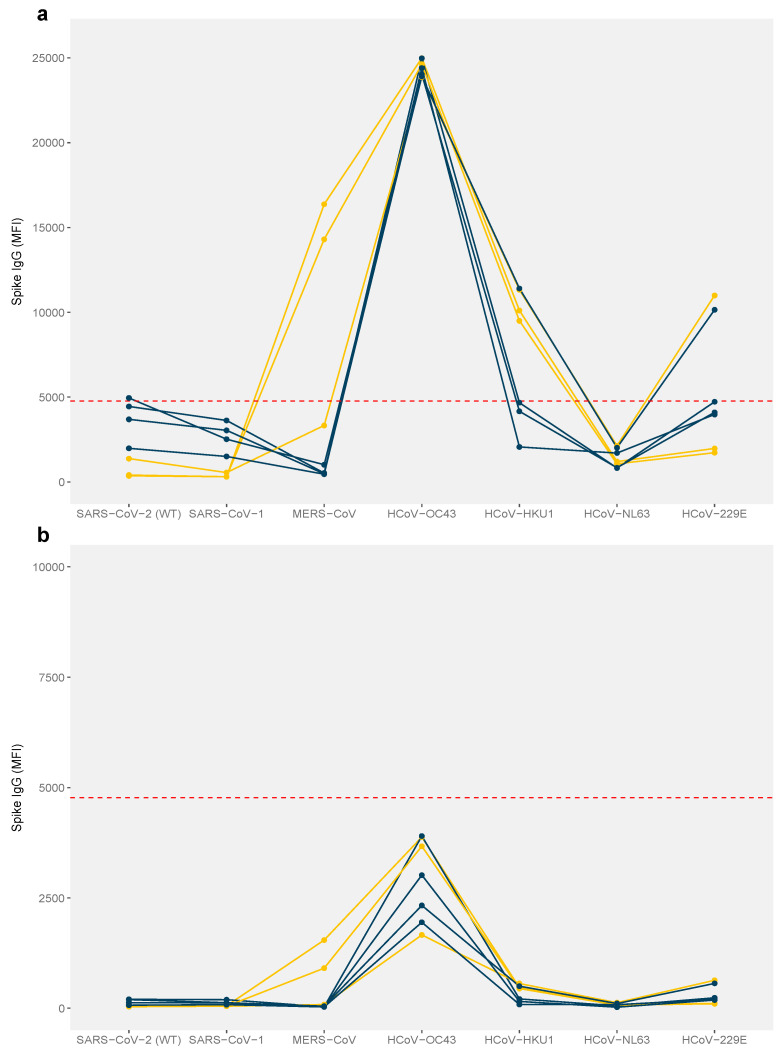
MFI levels of IgG antibodies in betacoronavirus-positive pre-pandemic samples (*n* = 7). (**a**) sera diluted at 1:400 and (**b**) at 1:8000 were tested. IgG levels (MFI) above 4774 indicate seropositivity (indicated by the red dashed line). Teal and yellow lines represent SARS-CoV-2 and MERS-CoV seropositive samples by the β-CoV MMIA, respectively.

Seven serum samples with antibodies reactive to SARS-CoV-2 and MERS-CoV from the β-CoV MMIA were subsequently tested using the SC2/HCoV MMIA, to further investigate cross-reactivity possibly associated with prior HCoV infection. All seven samples showed very strong binding to the HCoV-OC43 spike protein (above 23,000 MFI, approaching the machine upper quantification limit) at dilution 1:400 (Figure 3a); however, one serum sample remained weakly positive for SARS-CoV-2 with a slightly reduced MFI relative to that of the β-CoV assay. Additionally, two samples collected in 2017 and 2018 from one enrolled participant remained positive for MERS-CoV in the presence of the HCoV spike glycoproteins (Figure 3a). All the samples were seronegative when titered to 1:8000 (Figure 3b).

**Figure 4:**
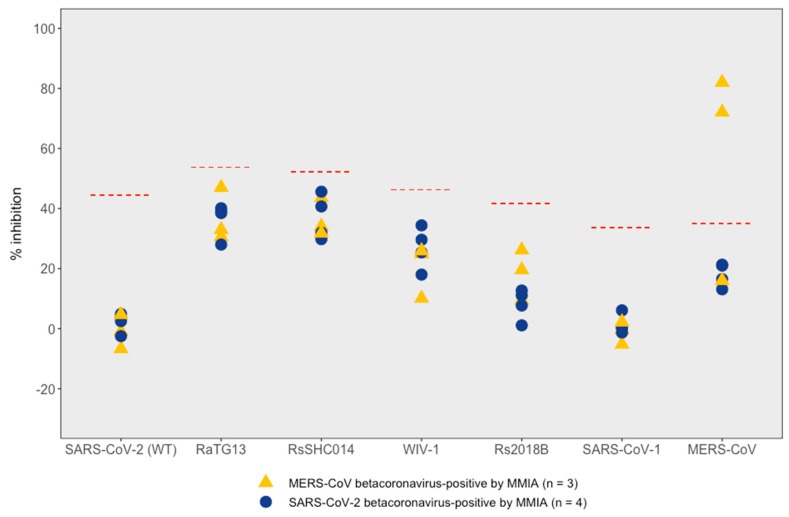
Multiplex sVNT assay of pre-pandemic betacoronavirus-positive samples (*n* = 7). Samples were diluted 1:320 and tested for sarbecovirus RBDs and MERS-CoV spike protein. The multiplex panel included six RBD antigens (SARS-CoV-2 ancestral strain, bat CoV RaTG13, bat CoV RsSHC014, bat CoV WIV-1, bat CoV Rs2018B and SARS-CoV-1) and one MERS-CoV spike (S1) antigen. Red dashed lines indicate the dynamic threshold cutoff for each antigen.

The β-CoV MMIA seroreactive samples (*n* = 7) were further screened for neutralization ability against three emerging human coronaviruses (SARS-CoV-1, SARS-CoV-2 and MERS-CoV) and four bat coronaviruses (RaTG13, WIV-1, Rs2018B and RsSHC014) using a multiplex surrogate virus neutralization (sVNT) assay. At dilution 1:320, all serum samples were negative for SARS-CoV-1 and SARS-CoV-2 RBDs, while two samples from the same individual showed sVNT antibodies reactive to MERS-CoV S1 above the threshold cutoff, with 81.97 and 72.11 percent inhibition of samples collected in 2017 and 2018, respectively (Figure 4, Table S4). The multiplex sVNT results of SARS-CoV-2, SARS-CoV-1 and MERS-CoV were consistent with the SC2/HCoV MMIA results (Table S1).

### 3.3. Post-COVID-19 Emergence Cohort

To investigate the cross-reactivity of SARS-CoV-2 with presently described bat coronaviruses during the COVID-19 pandemic, an additional 128 serum samples were collected from 88 participants, including 40 with follow-up samples, at the same collection site as the pre-pandemic cohort. Samples were collected at two time points: November 2021(*n* = 55) and January 2023 (*n* = 73). The enrolled participants received different SARS-CoV-2 vaccination regimens (Table S5). The numbers of unvaccinated participants in 2021 and 2023 were the same (*n* = 7). The majority of the enrolled participants received two doses of vaccines: 60.00% and 72.60% in 2021 and 2023, respectively.

**Figure 5:**
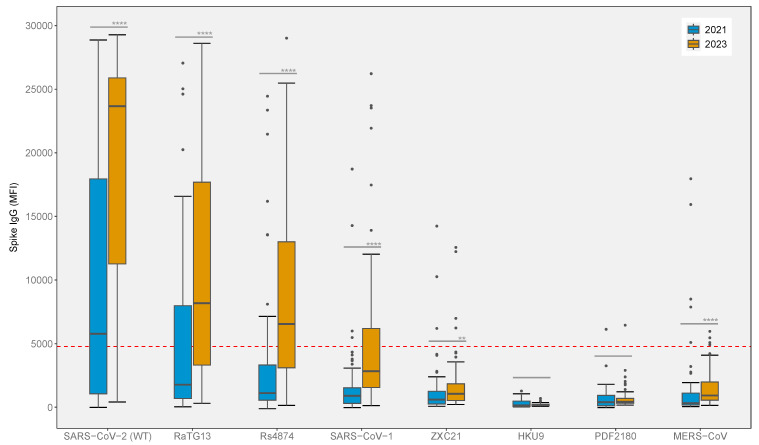
β-CoV MMIA serological screening of post-COVID-19 emergence cohort (*n* = 128). Serum samples collected in 2021 (*n* = 55) and 2023 (*n* = 73) were tested at 1:400 dilution for spike IgG reactivity against betacoronavirus. MFI values above the threshold cutoff of 4774 MFI (red dashed line) were considered seropositive. The unpaired Mann-Whitney U test was used to compare each targeted antigen’s MFI between 2021 and 2023. **** *p*-values < 0.0001, ** *p*-values < 0.01.

The spike IgG reactivity against betacoronaviruses of serum samples collected in 2021 and 2023 were tested using the β-CoV MMIA. We observed a significant IgG cross-reactivity between SARS-CoV-2 WT, RaTG13 and Rs4874 in serum samples collected in 2023 compared to 2021 (*p*-values ≤ 0.0001) with the median MFI levels above the threshold cutoff (Figure 5). The median MFI levels were 23,669 and 5774 for SARS-CoV-2 WT, 8180 and 1775 for RaTG13 and 6545 and 1116 for Rs4874 in 2023 and 2021, respectively. In addition, a higher positive rate of three antigens was shown in serum samples collected in 2023 compared to 2021: 83.56% and 54.55% for SARS-CoV-2, 67.12% and 30.91% for RaTG13 and 57.53% and 18.18% for Rs4874, respectively, highlighting the cross-reactivity induced by the administration of COVID-19 vaccines with or without SARS-CoV-2 infection. We observed a significant difference in median MFI for SARS-CoV, ZXC21 and MERS-CoV; however, the median MFI values were below the threshold cutoff (Figure 5).

**Figure 6:**
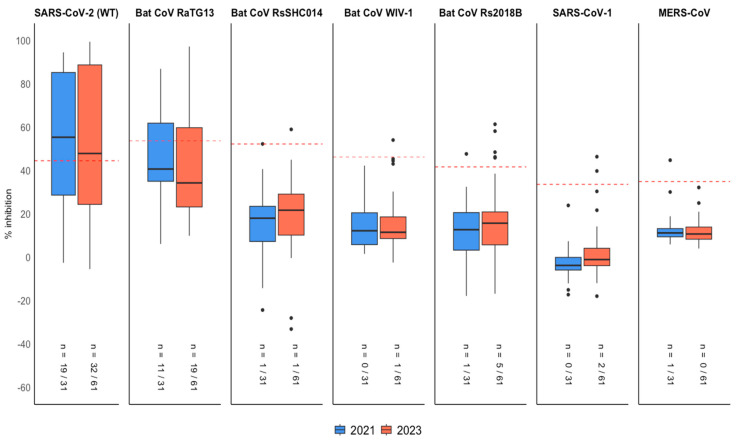
Multiplex sVNT assay of post-COVID-19 betacoronavirus-positive samples (*n* = 92). Serum samples collected in 2021 (*n* = 31) and 2023 (*n* = 61) were diluted 1:320 and tested for surrogate neutralizing antibodies against sarbecovirus RBDs and MERS-CoV spike (S1). Red dashed lines represent threshold cutoff values for each antigen.

Seropositive sera by the β-CoV MMIA collected in 2021 (*n* = 31) and 2023 (*n* = 61) were further tested using a multiplex sVNT to detect the total immunodominant surrogate neutralizing antibodies. A total of 20/31 (64.52%) showed surrogate neutralizing antibodies against betacoronaviruses above the designated cutoff at dilution 1:320, including SARS-CoV-2 (19/31, 61.29%), RaTG13 (11/31, 35.48%), Rs2018B (1/31, 3.23%), RsSHC014 (1/31, 3.23%) and MERS-CoV (1/31, 3.23%), by multiplex sVNT. A lower positive percentage of SARS-CoV-2 (32/61, 52.46%) was observed in the 2023 serum samples (*n* = 61). These samples demonstrated a cross-reactivity of SARS-CoV-2 and other sarbecoviruses, including 19/61 (31.15%) to RaTG13, 1/61 (1.64%) to RsSHC014, 1/61 (1.64%) to WIV-1, 5/61 (8.20%) to Rs2018B and 2/61 (3.28%) to SARS-CoV-1 (Figure 6).

### 3.4. PCR Results

To confirm the evidence of coronavirus infection of enrolled participants, coronavirus family-wide PCR was performed from oral, nasal or nasopharyngeal swabs collected on the same day as blood samples [19]. Among a total of 228 pre-pandemic samples, all samples were found to be negative [11], while seven nasopharyngeal swabs (7/73, 9.59%) collected during the COVID-19 pandemic in 2023 were detected as positive for SARS-CoV-2 (4/73, 5.48%), HCoV-OC43 (1/73, 1.37%) and HCoV-229E (2/73, 1.37%) (Table 3).

## 4. Discussion

Occupational exposure to wildlife is understood to be a major factor in disease zoonosis. Bat guano collectors are perceived to be vulnerable to coronavirus spillover from bats; thus, it is crucial to quantify the potential risks associated with this occupational activity. At our study site, guano collectors work within a bat cave once a week (3–4 h) and have close contact with bats and bat products (feces, urine and bat carcasses), but report limited personal protective equipment (PPE) use. At least 14 bat species, including *R. pusillus*, have been detected roosting within the cave. Bat-borne sarbecoviruses closely related to SARS-CoV-2 have been identified in *R. pusillus* in China and Laos [7,20]. Additionally, a MERS-like CoV was detected by PCR in dry bat guano at a cave in the Khao Chong Phran Non-hunting Area in Ratchaburi Province [10]. In this study, blood samples were collected from bat guano collectors at four time points in 2017, 2018, 2021 and 2023. The samples were used to investigate antibodies against betacoronavirus in the pre- and post-SARS-CoV-2 eras.

We detected anti-spike antibodies against SARS-CoV-2 in four pre-pandemic serum samples using an exploratory β-CoV MMIA (Figure 2 and Figure S1). These samples were further tested for common human coronaviruses (HCoVs) in a well-qualified SC2/HCoV MMIA [14], and we detected no reactivity with SARS-CoV-2 spike binding antibodies when HCoV spike antigens were included in the multiplex panel (Figure 3). In contrast to the first β-CoV MMIA results, all the tested samples showed strong reactivity for HCoV-OC43, suggesting that the initial detection of anti-SARS-CoV-2 IgG likely resulted from the pre-existing cross-reactivity induced by prior HCoV-OC43 infections. As previously reported, pre-pandemic serums, including those with PCR-confirmed HCoV infection, tested in the absence of HCoV antigens reported elevated SARS-CoV-2 reactivity [14]. However, the inclusion of HCoV antigens reduced cross-reactive binding to SARS-CoV-2 spike, thus enhancing assay specificity. Our results follow a similar pattern, where SARS-CoV-2 reactivity was reduced below the positivity threshold when tested alongside the HCoV antigens.

Moreover, the four serum samples underwent additional testing to detect neutralizing antibodies against SARS-CoV-2 and other betacoronaviruses in the multiplex sVNT. All samples tested negative for multiplex sVNT (Figure 4), confirming the absence of serologic evidence for ACE-2-using coronaviridae (RaTG13, RsSHC014, WIV-1, CoV Rs2018B) in the bat guano collectors prior to the COVID-19 pandemic and providing an important confirmatory test of pre-SARS-CoV-2 seronegativity. Our findings are consistent with other studies that detected the serological cross-reactivity of HCoVs with SARS-CoV-2 spike, RBD and nucleocapsid (NC) proteins in the pre-pandemic serum samples [21,22,23]. This cross-reactivity is likely to be driven by the exposure to betacoronaviruses that share spike protein structure with SARS-CoV-2, including HCoV-OC43 and HCoV-HKU1, which share approximately 40% sequence similarity with multiple highly conserved regions [13]. Our neutralizing antibody results were consistent with other studies demonstrating that pre-existing SARS-CoV-2 cross-reactive binding antibodies induced by prior HCoV infections do not neutralize SARS-CoV-2 [13,24]. Our study stands in contrast to other pre-COVID-19 studies such as one that examined samples collected between 2017 and 2020 and reported a sarbecovirus seroprevalence of 33.3% among individuals working in extractive industries, including bat guano harvesting [15].

Common HCoVs typically cause the common cold and represent approximately 10% of mild to moderate upper and lower respiratory tract infections globally. Among the four common HCoVs, HCoV-OC43 was the most frequently detected, followed by HCoV-NL63, HCoV-HKU1 and HCoV-229E [25]. Infections of HCoV-OC43 were observed in the majority of children and the seroconversion has been observed early in life [26]. In Thailand, common HCoVs were reported in multiple studies on patients and healthy individuals among multiple communities. A study in eastern Thailand detected up to 70% of HCoV-OC43 infection in patients with HCoV infections who were enrolled in active surveillance for pneumonia requiring hospitalization [27]. Similarly, HCoV-OC43 was reported to be the predominant HCoV detected in outpatients with influenza-like illness in southern Thailand [28]. Furthermore, HCoV-HKU1 was detected in a nasopharyngeal swab collected from an asymptomatic individual who regularly collected bat guano in a cave in Ratchaburi province [29]. A prior serological survey was conducted in 10 provinces in Thailand, and approximately 10% HCoV seropositivity was detected in the pre-pandemic serum samples screened by ELISA, with a 4.75% odds ratio for HCoV seropositivity in participants with bat exposure compared to the non-exposure group [30]. These findings provide evidence of the circulation of HCoVs among patients with respiratory illness and healthy individuals exposed to bats or bat excretions in the communities in Thailand. We recommend including four common HCoVs and other emerging CoVs in the serological testing panel to eliminate any cross-reactivity and reduce false positive results.

The vaccine types administered to the general Thai population in 2021 and 2023 followed mixed regimens, including inactivated virus vaccines, viral vector vaccines, and mRNA vaccines. Following COVID-19 vaccination, the number of participants with neutralizing antibodies against RaTG13 increased significantly compared to the pre-pandemic cohort. Among the SARS-CoV-2 seroreactive samples, either from infection or vaccination, collected in 2021 (19/31, 61.29% positivity) and 2023 (32/61, 52.46% positivity), 57.89% (11/19) and 59.38% (19/32), respectively, were also seropositive for RaTG13 (Figure 6). All participants who tested positive for RaTG13 antibodies had a history of vaccination and/or previous COVID-19 infection. The identity and similarity percentages between the SARS-CoV-2 spike protein and the RaTG13 spike protein are 97.4% and 98.4%, respectively, highlighting a significant genetic relationship between the two coronaviruses as well as conserved receptor-binding ability [31]. Previous studies have shown that antibodies generated by SARS-CoV-2 vaccination can exhibit cross-reactivity and partial cross-protection with bat coronaviruses such as RaTG13 [16,32,33].

We report the detection of anti-MERS-CoV spike antibodies in an occupationally exposed human population with frequent contact with bats and/or bat products. Notably, only one participant (1/237, 0.43%), who reported their primary livelihood as a forest ranger, exhibited both total binding antibodies and surrogate neutralizing antibodies against MERS-CoV as detected by MMIA and multiplex sVNT assays, respectively. Samples from this participant (*n* = 3) were collected in 2017, 2018 and 2021. However, due to the unavailability of standard confirmatory assays such as the plaque reduction neutralization test (PRNT) in our laboratory, further validation of these results could not be performed. It is important to note that serological cross-reactivity of MERS-CoV has been documented in the seroconversion of COVID-19 patients [34]. These serological cross-reactive antibodies are likely explained by the spike protein sequence homology between SARS-CoV-2 and MERS-CoV, with 58% similarity [13]. However, the presence of MERS-CoV antibodies in pre-COVID-19 pandemic samples in our study is unlikely to have resulted from cross-reactivity with SARS-CoV-2 antibodies, as SARS-CoV-2 antibodies were not detected (Figure 3a). Despite this, the finding remains inconclusive without additional confirmatory assays such as PRNT or virus isolation, and without the identification of a local animal reservoir of MERS-CoV.

The MERS-CoV-seropositive participant, who oversaw a bat cave and occasionally collected bat guano for fertilizer, reported no recent overseas travel or acute illness within the 12 months prior to enrollment during the pre-pandemic period. Additionally, swab samples from this participant tested negative for betacoronaviruses by Quan-CoV family-wide PCR at all time points (Table 3), therefore, viral sequencing could not be performed. The first MERS-CoV case in Thailand, detected in June 2015, was imported and did not result in localized transmission [35]. This, in combination with the absence of antibodies in other participants, suggests that the seropositivity in this individual was not due to human-to-human transmission. We hypothesize that the detected antibodies resulted from exposure to a coronavirus antigenically related to MERS-CoV with the ability to bind the DPP4 MERS-CoV receptor. However, the proposed host species of MERS-CoV, *Taphozous perforates* (Egyptian tomb bat), has not been documented in Thailand [36]. Future research should prioritize serological surveillance of betacoronaviruses in bats and the discovery of the hypothesized MERS-like-CoV genomes with subsequent infectivity and pathogenicity characterization to elucidate zoonotic transmission mechanisms and identify potential spillover threats.

A potential limitation of this study is that the targeted bat-borne antigens used to capture antibodies may not perfectly match the betacoronaviruses detected in this cave. However, the β-CoV MMIA was specifically designed to detect a broad range of coronaviruses, including novel related coronaviruses, by using the whole spike proteins to detect binding antibodies.

To minimize the risk of zoonotic transmission, it is crucial to continue monitoring the high-risk populations for potential bat coronavirus spillover. This should be done alongside efforts to improve the environment and encourage proper practices among villagers, such as wearing appropriate PPE to reduce the possibility of bat-to-human viral transmission. 

## Figures and Tables

**Table 1:** Enrollment cohort and collected samples, including serum and swab samples.

Number of Enrolments	Number of Participants	Gender		Number of Collected Samples			
		Female	Male	2017	2018	2021	2023
Single	159	87	72	63	66	7	23
Multiple enrollments							
Two	49	28	21	26	26	20	26
Three	17	4	13	12	11	16	12
Four	12	5	7	12	12	12	12
Total	237	124	113	113	115	55	73

**Table 2:** Demographic characteristics of study populations across four years (2017–2023).

Characteristics	Pre-COVID-19 Pandemic		Post-SARS-CoV-2 Emergence	
	2017 (*n* = 113)	2018 (*n* = 115)	2021 (*n* = 55)	2023 (*n* = 73)
Gender				
Female	57 (50.44%)	70 (60.87%)	18 (32.73%)	30 (41.10%)
Male	56 (49.56%)	45 (39.13%)	37 (67.27%)	43 (58.90%)
Age group				
12–17 years	3 (2.65%)	4 (3.48%)	0 (0.00%)	1 (1.37%)
18–35 years	24 (21.24%)	19 (16.52%)	7 (12.73%)	11 (15.07%)
36–55 years	53 (46.90%)	47 (40.87%)	27 (49.09%)	35 (47.95%)
>55 years	33 (29.20%)	45 (39.13%)	21 (38.18%)	26 (35.62%)
Median (range)	47 (14–82)	50 (12–89)	52 (23–99)	52 (16–91)
Residence time				
<1 year	4 (3.54%)	2 (1.74%)	1 (1.82%)	1 (1.37%)
1–5 years	6 (5.31%)	3 (2.61%)	3 (5.45%)	4 (5.48%)
6–10 years	45 (39.82%)	3 (2.61%)	3 (5.45%)	7 (9.59%)
>10 years	58 (51.33%)	107 (93.04%)	48 (87.27%)	61 (83.56%)
Primary livelihood				
Crop Production	22 (19.47%)	17 (14.78%)	6 (10.91%)	10 (13.70%)
Forest ranger	13 (11.50%)	13 (11.30%)	11 (20.00%)	14 (19.18%)
Bat guano harvesting	30 (26.55%)	22 (19.13%)	18 (32.73%)	15 (20.55%)
Other	46 (40.71%)	63 (54.78%)	18 (32.73%)	33 (45.21%)
Unemployed/retired	2 (1.77%)	0 (0.00%)	2 (3.64%)	1 (1.37%)
Animal contact last year				
Scratched/bitten by animal	34 (30.09%)	9 (7.83%)	14 (25.45%)	10 (13.70%)
Contact with rodents	12 (10.62%)	18 (15.65%)	16 (29.09%)	13 (17.81%)
Contact with bats/bat products	13 (11.50%)	4 (3.48%)	43 (78.18%)	51 (69.86%)
Contact with non-human primates	1 (0.88%)	1 (0.87%)	10 (18.18%)	8 (10.96%)
COVID-19 vaccination	Not Applicable	Not Applicable	48 (87.27%)	66 (90.41%)
COVID-19 history of infection	Not Applicable	Not Applicable	0 (0.00%)	28 (38.36%)

**Table 3:** PCR results of coronavirus detection from oral (OS), nasal (NS) or nasopharyngeal (NPS) swabs using Quan-CoV family-wide PCR.

Year of Enrolment	Specimen Type	Number of Samples		Detected Coronavirus * (Number)
		Tested Samples	Positive Samples (%)	
2017	OS, NPS	113	0	Not applicable
2018	NS	115	0	Not applicable
2021	NPS	55	0	Not applicable
2023	NPS	73	7 (9.59%)	SARS-CoV-2 (4)
				HCoV-OC43 (1)
				HCoV-229E (2)

* The detected virus species was identified by Sanger sequencing from PCR-positive samples.

## Data Availability

Data are available from the authors upon reasonable request and with permission of Supaporn Wacharapluesadee and Opass Putcharoen to any researcher wishing to use them for non-commercial purposes. Researchers who wish to obtain a copy of the data may submit their request to supaporn.wac@chula.ac.th and opassid@gmail.com.

## Supplementary Materials

The following supporting information can be downloaded at: https://www.mdpi.com/article/10.3390/v17060837/s1, Table S1: Pre-pandemic serum samples positive by the β-CoV MMIA and further tested using (a) SC2/HCoV MMIA and (b) multiplex sVNT. Color coding indicates results above the designated threshold cutoffs; Table S2: Characteristics of bat guano collectors routinely exposed to bat products through occupational activities (not necessarily primary livelihood); Table S3: β-CoV MMIA testing results from serum samples collected from a single study site over a four-year period; Table S4: Comparison of MERS-CoV seroprevalence between the β-CoV MMIA and multiplex sVNT for all serum samples; Table S5: SARS-CoV-2 seropositivity among participants enrolled during the COVID-19 pandemic in 2021 and 2023, as determined using the β-CoV MMIA; Figure S1: β-CoV MMIA testing results across four study years (2017–2023). Red dashed line indicates the MFI threshold cutoff.

## Disclaimer

Eric D. Laing is a federal employee within the Department of Defense. This work was prepared as part of their official duties. Title 17, U.S.C., §105 provides that copyright protection under this title is not available for any work of the U.S. Government. Title 17, U.S.C., §101 defines a U.S. Government work as a work prepared by an employee of the U.S. Government as part of that person’s official duties. The views expressed in the article are those of the authors and do not necessarily express the official policy and position of the Uniformed Services University of Health Sciences, Henry M. Jackson Foundation for the Advancement of Military Medicine, the Department of Defense, the U.S. government, or any of the other institutions affiliated with any of the authors.

## Abbreviations

The following abbreviations are used in this manuscript:

MMIA	Multiplex microsphere immunoassay
Multiplex sVNT	Multiplex surrogate virus neutralization test
HCoV(s)	Human coronavirus(es)
