# Supplementary material for: Serological Surveillance of Betacoronaviruses in Bat Guano Collectors: Pre-COVID-19 Pandemic and Post-SARS-CoV-2 Emergence
Source: Viruses. 2025 Jun 10;17(6):837. doi: 10.3390/v17060837 (PMC12197454; doi:10.3390/v17060837)
Supplement: Supplementary file 1 [file viruses-17-00837-s001.zip › viruses-3596453-supplementary.pdf]

Table S1. Pre-pandemic serum samples positive by the β-CoV MMIA and further tested using (a) SC2/HCoV MMIA and (b) multiplex sVNT. Color coding indicates results above the designated threshold cutoffs.

a

#ID	Year of enrollment	The β-CoV MMIA positive	The SC2/HCoV MMIA (MFI) at dilution 1:400						
			SARS-CoV-2	SARS-CoV-1	MERS-CoV	HCoV-OC43	HCoV-HKU1	HCoV-NL63	HCoV-229E
1	2017	MERS-CoV	407	308	16368	24977	10105	1197	1970
2	2017	SARS-CoV-2	4451	3625	528	24969	4673	824	4723
3	2018	MERS-CoV	355	303	14297	24622	9497	1068	1722
4	2018	MERS-CoV	1371	555	3324	23865	11326	2130	10990
5	2018	SARS-CoV-2	4952	2524	1015	23907	11399	2019	10147
6	2018	SARS-CoV-2	1983	1502	457	24394	2059	1709	3976
7	2018	SARS-CoV-2	3689	3038	479	24022	4161	833	4101

b

#ID	Year of enrollment	The β-CoV MMIA positive	sVNT (% inhibition) at dilution 1:320						
			SARS-CoV-2	SARS-CoV-1	MERS-CoV	RaTG13	RsSHC014	WIV-1	Rs2018B
1	2017	MERS-CoV	-6.74	-5.18	81.97	47.01	43.56	24.93	9.47
2	2017	SARS-CoV-2	4.1	0.45	21.28	40.07	45.63	34.42	7.71
3	2018	MERS-CoV	-0.86	-0.45	72.11	30.62	34.09	10.08	19.64
4	2018	MERS-CoV	4.66	2.17	15.97	33.01	31.81	25.85	26.15
5	2018	SARS-CoV-2	4.91	6.08	16.47	38.52	29.76	18.01	11.07
6	2018	SARS-CoV-2	-2.48	0.17	13.07	39.23	31.99	25.43	12.73
7	2018	SARS-CoV-2	2.54	-1.29	20.98	27.99	40.66	29.57	1.08

Table S2. Characteristics of bat guano collectors routinely exposed to bat products through occupational activities (not necessarily primary livelihood).

Characteristics	Bat guano collectors	
	2021 (*n* = 28)	2023 (*n* = 38)
**Distance from home to the bat cave**		
<5 km	27 (96.43%)	37 (97.37%)
5-10 km	1 (3.57%)	1 (2.63%)
**Time working at the guano collection site**		
<1 year	6 (21.43%)	4 (10.53%)
1-5 years	8 (28.57%)	10 (26.32%)
>5 years	14 (50.00%)	24 (63.16%)
**Working frequency**		
Once a week	27 (96.43%)	38 (100.00%)
A few times a year	1 (3.57%)	0 (0.00%)
**Length of contact with bat products**		
15 mins	3 (10.71%)	1 (2.63%)
30 mins	1 (3.57%)	0 (0.00%)
>1 hour	24 (85.71%)	37 (97.37%)
**Personal Protective Equipment**		
Face masks	20 (71.43%)	20 (52.63%)
Face covers/ parts of t-shirt stretched to the face	21 (75.00%)	30 (78.95%)
Hat	9 (32.14%)	8 (21.05%)
Gloves	14 (50.00%)	16 (42.11%)
Long sleeve shirts/ over shirts	15 (53.57%)	25 (65.79%)
Long pants	13 (46.43%)	25 (65.79%)
No protection	0 (0.00%)	1 (2.63%)

Table S3. β-CoV MMIA testing results from serum samples collected from a single study site over a four-year period.

Year	MMIA results	SARS-CoV-2 (WT)	bat CoV RaTG13	bat CoV Rs4874 (WIV16)	SARS-CoV-1	bat CoV ZXC21	bat CoV HKU9	bat CoV PDF-2180	MERS-CoV
2017 (*n* = 113)	Positive rate	1 (0.88%)	0 (0.00%)	1 (0.88%)	0 (0.00%)	0 (0.00%)	0 (0.00%)	0 (0.00%)	1 (0.88%)
	median	1133	649	759	1091	590	708	931	802
	range	75-5188	84-2720	-64-4997	50-3866	92-2273	88-2784	87-2344	73-19269
2018 (*n* = 115)	Positive rate	3 (2.61%)	1 (0.87%)	1 (0.87%)	1 (0.87%)	0 (0.00%)	0 (0.00%)	0 (0.00%)	2 (1.74%)
	median	1261	668	781	1127	662	760	962	910
	range	55-9493	91-5381	-49-9446	41-6454	85-4774	85-1983	81-2891	84-11855
2021 (*n* = 55)	Positive rate	30 (54.55%)	17 (30.91%)	10 (18.18%)	4 (7.27%)	3 (5.45%)	0 (0.00%)	1 (1.82%)	5 (9.09%)
	median	5774	1775	1116	900	598	142	400	316
	range	-10-28868	32-27051	-111-24449	-30-18722	71-14237	23-1275	-25-6128	49-17962
2023 (*n* = 73)	Positive rate	61 (83.56%)	49 (67.12%)	42 (57.53%)	28 (38.36%)	4 (5.48%)	0 (0.00%)	1 (1.37%)	5 (6.85%)
	median	23669	8180	6545	2835	1058	155	430	923
	range	413-29276	304-28614	142-29013	125-26222	216-12563	67-694	169-6448	148-5966

Table S4. Comparison of MERS-CoV seroprevalence between the β-CoV MMIA and multiplex sVNT for all serum samples.

Year of enrollment	MERS-CoV Seroprevalence	
	The β-CoV MMIA (1:400)	Multiplex sVNT (1:320)
2017 (*n* = 113)	1 (0.88%)	1 (0.88%)
2018 (*n* = 115)	2 (1.74%)	1 (0.87%)
2021 (*n* = 55)	5 (9.09%)	1 (1.82%)
2023 (*n* = 73)	5 (6.85%)	0 (0.00%)

Table S5. SARS-CoV-2 seropositivity among participants enrolled during the COVID-19 pandemic in 2021 and 2023, as determined using the β-CoV MMIA.

Number of COVID-19 vaccine doses	No. of SARS-CoV-2 positive/ no. of tested (%)	
	2021 (*n* = 55)	2023 (*n* = 73)
Unvaccinated	0/7 (0.00%)	1/7 (14.29%)
One	1/14 (7.14%)	2/3 (66.67%)
Two	28/33 (84.85%)	48/53 (90.57%)
Three	1/1 (100.00%)	9/9 (100.00%)
Four	0/0 (0.00%)	1/1 (100.00%)

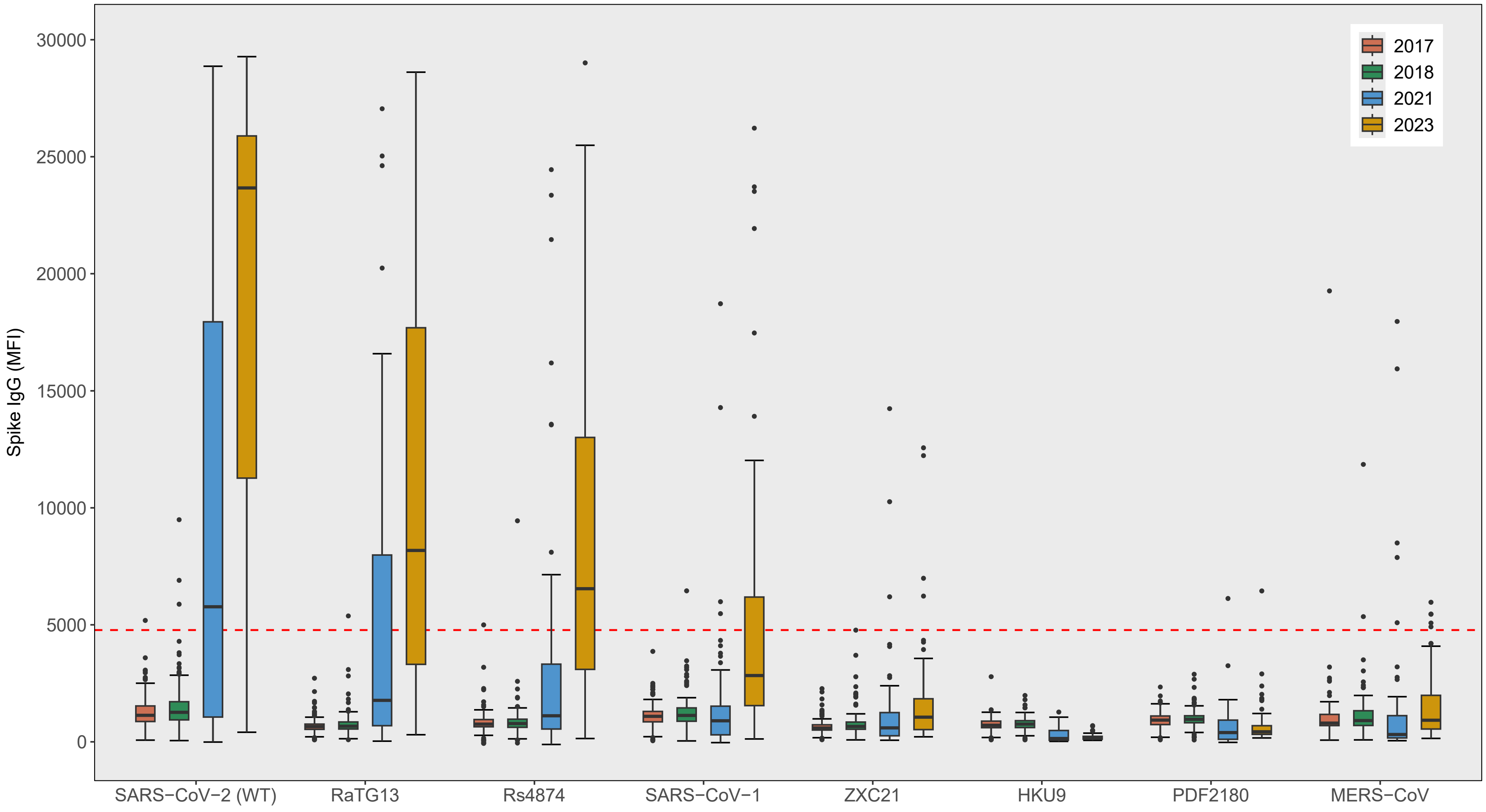

Figure S1. β-CoV MMIA testing results across four study years (2017-2023). Red dashed line indicates the MFI threshold cutoff.
